# Professional experiences of accessing cancer drugs in the NHS: the introduction of the Cancer Drugs Fund, the individual versus the population

**DOI:** 10.1186/1472-6963-14-S2-O41

**Published:** 2014-07-07

**Authors:** Charlotte Chamberlain, Amanda Owen-Smith, William Hollingworth, Jenny Donovan

**Affiliations:** 1 School of Social and Community Medicine, University of Bristol, Bristol, UK

## Background

The establishment of the Cancer Drugs Fund (CDF), which removed the necessity for cost-effectiveness as a criterion for drugs access and expedited access to non-NICE appraised drugs, has significantly changed prescribing of cancer drugs for terminally ill cancer patients in the NHS. Neither clinicians nor the public have a preference for spending money on cancer over other disease states. The most effective and efficient way to spend resource within cancer care is hotly debated.

## Materials and methods

Audio-recorded, semi-structured interviews with oncology and palliative care consultants (n=16) took place in the South West region (March-October 2013). Transcripts were analyzed iteratively using constant comparison to identify and understand common themes. Matrices were developed to explore and compare derived themes and highlight dissonant views.

## Results

The complex dynamic of personal, familial, media and political influence on palliative chemotherapy decision-making were described by participants. Decision-making around palliative chemotherapies was improved by the CDF according to all oncology participants. The main benefit of the CDF was thought to be access to treatments not previously available to patients. However, the improved availability of high cost drugs caused conflict for clinicians reconciling the apparent benefits for the patient and the cost for the population. The disadvantages of the CDF included a potential reduction in clinician discretion in chemotherapy decision-making since the National CDF Cohort List and a delay in advanced care planning conversations. The appropriateness of ‘weighting’ the value of life more at the end of life (as defined by NICE) was felt to be artificial by 5/6 palliative care and 5/10 oncology consultants. The majority of consultants described an uncomfortable compromise between the importance of that time to some individuals offset by the lessened quality of life for the majority (13/16). Professional opinion clashed most over the role of Multi-Disciplinary Team meetings (MDTs) in accessing CDF drugs. MDTs were felt to be a cause for delay and ‘a tick-box’ exercise (oncologists) and a missed opportunity for patient advocacy (palliative care).

## Conclusions

Clinicians indicated that they mostly approved of the CDF in enabling patient access to new palliative chemotherapy. However; clinicians sometimes struggled with judging the balance between potential gains and losses in length and quality of life for individual patients and the level of financial cost incurred by the NHS. The views of patients accessing drugs through the CDF are currently being sought in order to improve understanding of the impact of the CDF.

